# Quinine in Fever

**Published:** 1857-09

**Authors:** 


*Quinine in Fever. By Isaac Casselberry, M. D., of Evansville, Indiana.*

This is a pamphlet of 13 pages, reprinted from the Cincinnati *Medical Observer.* The writer says: "The first pathological state in the incipiency of fever is a lesion of automatic innervation, which produces a lesion of secretion and nutrition. The effete elements of the food and the transformed tissues are retained in the blood; both nutrition and secretion are decreased or perverted." Quinine he regards not as a stimulant in the same sense that we regard alcohol or the various fermented and distilled liquids, but an agent peculiarly adapted to increase and sustain the function of the *automatic* nervous system, and thereby counteract or remove the primary pathological lesion in febrile diseases, The article is well written and interesting, but the ideas contained in it can scarcely be regarded as new or original.

